# Clinical Value Screening, Prognostic Significance, and Key Gene Identification of TrkB in Laryngeal Carcinoma

**DOI:** 10.1155/2022/1354005

**Published:** 2022-08-19

**Authors:** Jing Zhang, Xiaoyan Hu, Shuang Liu, Yuxiao He, Liangge Lyu, Liang Jiang

**Affiliations:** ^1^Department of Otolaryngology Head and Neck Surgery, Affiliated Hospital of Southwest Medical University, Luzhou 646000, China; ^2^Department of Pathogen Biology, School of Basic Medicine Southwest Medical University, China; ^3^Public Center of Experimental Technology of Pathogen Biology Technology Platform Southwest Medical University, Luzhou 646000, China

## Abstract

**Purpose:**

Using human gene chip expression profiling technology to screen out downstream genes related to TrkB regulation in laryngeal cancer cells.

**Methods:**

Using the Hep-2 TrkB shRNA cell line, divide it into an experimental group (shNTRK2) and a control group (PLKO1), and use the human gene expression microarray to screen out the differential genes. Then, select 10 upregulated genes and 10 downregulated genes from the differential genes, and use RT-PCR to verify whether the screening results of human gene expression microarray profiles are reliable. Use GO, KEGG, and miRNA enrichment analyses, PPI network diagram, etc., to analyze the differential genes and further screen out the key genes.

**Results:**

A total of 318 differential genes (87 upregulated genes and 231 downregulated genes) were screened in laryngeal cancer cells. Use RT-PCR for the 10 upregulated differential genes (DMKN, FHL1, FOXN4, GGNBP1, HOXB9, ABCB1, TNFAI, RGS2, LINC01133, and FGG) and 10 downregulated differential genes (CHI3L1, FMOD, IGFBP1, IRF5, SPARC, NPAS4, TRPS1, TRAP, COL8A1, and DNER), and the results are consistent with the chip results, confirming the accuracy of the chip results; GO analysis results show that the downstream differential genes (DEGs) regulated by TrkB are mainly involved in biological processes such as retinol metabolic process, diterpenoid metabolic process, and regulation of cell-substrate adhesion. DEGs mainly affect cytoskeletal protein binding, serotonin-activated cation-selective channel activity, and sphingosine molecular functions. DEGs are mainly enriched in the cell periphery, secretory granule, cytoplasmic membrane-bounded vesicle lumen, blood microparticle, and other molecular components. The results of disease enrichment analysis show that the downstream differential genes regulated by TrkB are mainly involved in atypical hemolytic uremic syndrome, hematologic disease, meningococcal disease, lung cancer, susceptibility, asthma, and other diseases. The PPI network diagram results showed 7 hub genes, and then, we used GO analysis and KEGG enrichment analysis to see the biological process, cell component, molecular functions, and biological pathways.

**Conclusion:**

Gene chip technology was used to screen out the differential genes of TrkB epigenetic modification in the Hep-2 cell line, and seven key genes (ALDH1A1, SDR16C5, PIK3R1, PLCG2, IL2RG, PIK3CD, and SPARC) were further screened using bioinformatics technology.

## 1. Introduction

Laryngeal carcinoma is a malignant tumor that occurs in the larynx, accounting for 25%-30% of all head and neck cancer cases, and is the most common solid cancer. More than 95% of laryngeal cancers are squamous cell carcinomas. Epidemiological investigations have shown that the incidence of laryngeal cancer is 2.1/100,000, which is a greater threat to human health [1–3]. Research results show that the occurrence of laryngeal cancer is related to a variety of factors. Smoking and drinking are positively related to the incidence of laryngeal cancer. Secondly, factors such as sulfur dioxide, arsenic, industrial dust, toxic chemicals, and HPV infection can all become incentives for laryngeal cancer [4, 5]. The main clinical manifestations of laryngeal cancer are hoarseness, dyspnea, coughing, dysphagia, cervical lymph node metastasis, etc. The order of appearance of symptoms at different primary sites is different [6–8]. Pathological biopsy under laryngoscopy is the gold standard for the diagnosis of laryngeal cancer. Combined treatments such as surgical treatment, radiotherapy, chemotherapy, and biological therapy have improved the survival rate of patients, but their curative effect is still not ideal [9, 10].

Tyrosine kinase receptor B (TrkB) is a type of protein kinase with multiple biological functions encoded by the NTRK2 gene. It is also a specific binding receptor for brain-derived nerve growth factors [11, 12]. TrkB can interact with BDNF. Specific binding, TrkB is activated by inducing dimerization of TrkB and induces its autophosphorylation, thereby exerting a series of biological effects [13–15]. Many studies have shown that TrkB participates in the regulation of tumor proliferation, invasion, and migration, but the expression and mechanism of TrkB in laryngeal cancer have not been studied [16–18]. The previous research results of our group showed that the expression of TrkB mRNA and protein was overexpressed in tumor tissues, and the high expression of TrkB significantly reduced the overall survival time of patients, which is closely related to clinical staging, lymph node metastasis, and smoking history, which can be an independent prognostic indicator for laryngeal cancer. However, whether TrkB can affect the expression of downstream genes through epigenetic modification, and the downstream genes that TrkB affects the metastasis of laryngeal cancer through epigenetic modification, still needs to be further clarified.

Therefore, this study used gene chip technology to screen out the downstream genes that were epigenetically modified by TrkB and then selected 10 upregulated differentially expressed genes and 10 downregulated differentially expressed genes, using RT-PCR to verify the correction.

## 2. Materials and Methods

### 2.1. Cell Grouping and Culture

Human Hep2 cell was purchased from China Center for Type Culture Collection. Then, we divided the cells into the experimental group (shNTRK2) and the control group (PLKO1) and place them in a 37°C 5% CO_2_ incubator. The culture medium is DMEM containing 10% fetal bovine serum (FBS), 1% penicillin, and streptomycin; replace the culture medium regularly.

### 2.2. Agilent Gene Chip Technology

Add nuclease-free water to 40 *μ*l in the labeled fluorescent DNA sample, then add 41.6 *μ*l hybridization solution, and centrifuge to mix. After detecting that the hybridization device is normal, add a 45 *μ*l hybridization solution and place the chip in the hybridization furnace for hybridization. Wash twice with washing solution I and washing solution II, each for 5 minutes. Use the steps in the Agilent chip scanner manual to scan the chip and save the hybridization picture. Use Agilent Feature Extraction (10.7) and CBC analyzer software to analyze and extract the data.

### 2.3. RT-PCR Verification of Differentially Expressed Genes

Ten genes that were upregulated and downregulated were selected from the differentially expressed genes to perform RT-PCR experiments for verification. First, extract the total RNA of the sample, reverse transcribed it into cDNA, and perform reverse transcription-polymerase chain reaction (RT-PCR) reaction. The reaction conditions are 95°C 5 min, 95°C 15 s, and 60°C 32 s, 40 cycles. Each sample was repeated 3 times.

### 2.4. Statistical Methods

The Gene Spring software V13 (Agilent) was used to summarize, standardize, and quality control the array data. The multiple of difference ≥ 1.5, *P* < 0.05, was used as the criteria for screening differential genes. The cluster 3.0 software was used for cluster analysis and principal component analysis. Use GO, KEGG, and other databases for bioinformatics analysis of differential genes.

## 3. Results

### 3.1. Quality Control Results of Gene Chip Data

**Figure 1:**
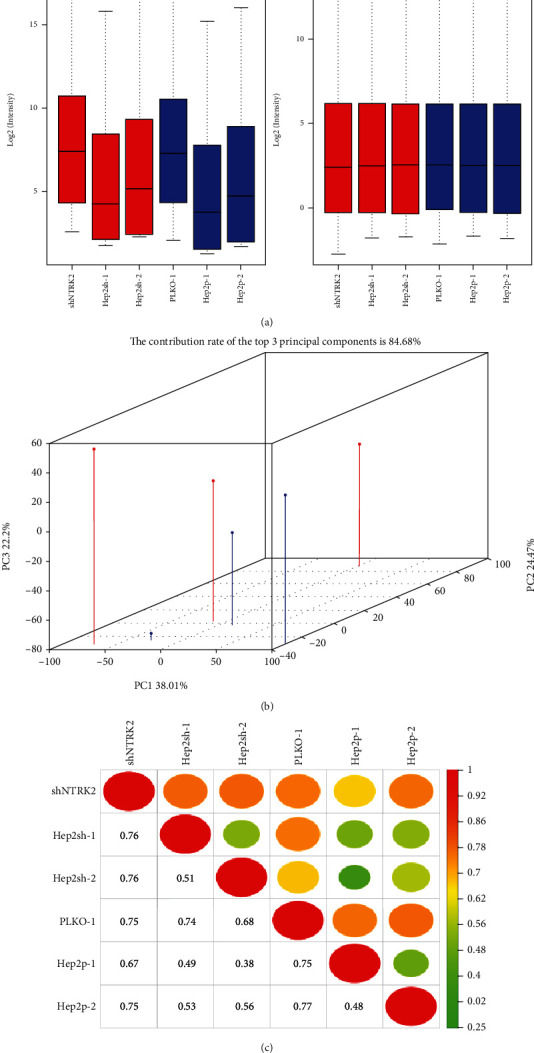
Quality control results of gene chip data. (a) Box plot for case vs. control. (b) The plot of PCA (each line represents a sample in the figure). (c) Pearson's correlation plot.

The box plot can visually display the gene expression of each sample before and after normalization. Red represents the experimental group, and blue represents the control group. From Figure 1(a), we can see that the distribution of all samples is relatively close, indicating that the repeatability of the chip between different samples tend to be consistent. Principal component analysis shows the distribution of all samples on the three main variables. Through principal component analysis, the data space is compressed, and the characteristics of multiple variables are visually displayed in the low-dimensional space. The PCA chart is used to indicate that each chip has a high degree of similarity within the group, but there is a certain difference between the groups. The results in Figure 1(b) show that the experimental group and the control group have good similarities, but the two groups also have big differences. The Pearson correlation coefficient graph shows the similarity of each sample. The closer the correlation coefficient is to 1.0, it means that the abscissa sample and the ordinate sample are positively correlated, and the negative correlation tends to be -1. The results in Figure 1(c) show that the correlation coefficients of the experimental group and the control group are both greater than 0.95, indicating that there is a high positive correlation between the 3 samples in the control group and the 3 samples in the experimental group.

### 3.2. Analysis Results of Gene Chip Data

#### 3.2.1. Differential Genes in Gene Chip Expression Profile

A gene chip experiment was performed after knocking down the TrkB gene in laryngeal cancer cells. The results showed that there were obvious differences in gene expression between the two groups. Using |LogFC| > 1.5, *P* < 0.05, as the screening criteria, a total of 318 differential genes were found, of which 87 belonged to highly expressed genes, and 231 belonged to low expressed genes. The top ten upregulated genes are DMKN, FHL1, FOXN4, GGNBP1, HOXB9, ABCB1, TNFAI, RGS2, LINC01133, and FGG; the top ten downregulated genes are CHI3L1, FMOD, IGFBP1, IRF5, SPARC, NPAS4, TRPS1 TRAP, COL8A1, and DNER.

#### 3.2.2. Difference Analysis of Expression Profile

**Figure 2:**
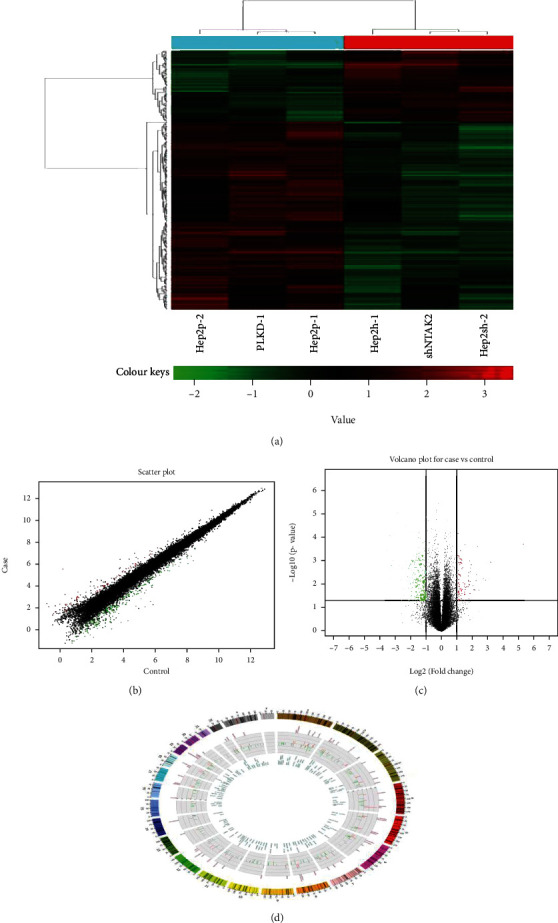
Expression profile difference analysis diagram. (a) The cluster diagram of genes expression profile. (b) Scatter plot for case vs. control. (c) Volcano plot for case vs. control. (d) Circos plot for case vs. control.

The results of the cluster analysis graph (Figure 2(a)) show that there is a significant difference in gene expression between the experimental group and the control group, and there is no significant difference in gene expression between the groups. As the abscissa and ordinate to draw a volcano map, the volcano map can directly reflect the significant difference between the two sets of sample data. The results of the volcano diagram (Figure 2(c)) show that there is a significant difference in gene expression between the experimental group and the control group. Downregulated genes account for the majority. Red indicates upregulated genes, green indicates downregulated genes, and black indicates genes with no significant difference. After standardizing the original chip data, a scatter plot is drawn. The scatter plot of the chip data is used to evaluate the central tendency of the overall distribution of the two sets of data. Each point in the scatter plot represents a probe point on the chip, which is in two dimensions. The position in the plane is determined by its abscissa and ordinate. The scatter plot (Figure 2(b)) of this experiment reflects the significant differences between the experimental group and the control group, where red represents upregulated genes, green represents downregulated genes, and black represents no significant differences in gene expression between different groups. The Circos diagram (Figure 2(d)) is based on gene location to show gene expression among different groups. The results show that there are significant differences between the experimental group and the control group. The outer circle represents the type of chromosome, and the inner circle represents the degree of difference in genes at that position. Red indicates upregulation of gene expression, green indicates downregulation of gene expression, and the length of the cylinder indicates the multiple of difference.

#### 3.2.3. Bioinformatics Analysis Results of Differentially Expressed Genes

**Figure 3:**
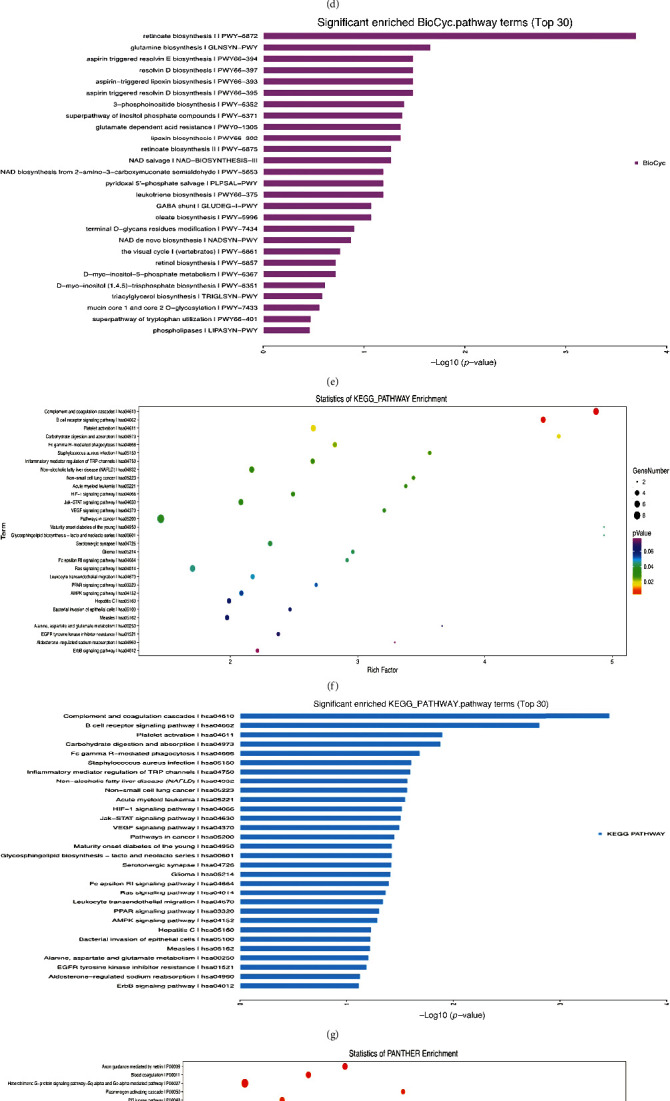
Differential gene signal pathway enrichment analysis diagram. (a) KEGG classification. (b) Statistics of pathway enrichment. (c) Significantly enriched pathway terms. (d) Statistics of Biocyc enrichment. (e) Significantly enriched Biocyc pathway terms. (f) Statistics of KEGG pathway enrichment. (g) Significantly enriched KEGG pathway terms. (h) Statistics of Panther enrichment. (i) Significantly enriched Panther pathway terms. (j) Statistics of Reactome enrichment. (k) Significantly enriched Reactome pathway terms.

*(1) Enrichment Analysis of Signal Pathways of Differential Genes*. After epigenetic modification of TrkB, we screened out 318 differential genes from 6 groups and analyzed the signal pathways of these differential genes through 6 databases (KEGG, PID, BioCarta, Reactome, Panther, and Biocyc) and obtained a significantly enriched signal pathway diagram (Figure 3). KEGG pathway enrichment analysis results show that differential genes are mainly involved: signal transduction, global and overview maps, immune system, platelet activation, pathway in cancer I hsa05200, complement and coagulation cascades I hsa04610, etc. Signal pathway is shown in Figures 3(a), 3(b), 3(f), and 3(g). Biocyc pathway enrichment analysis results show that differential genes are mainly involved in signal pathways such as retinoate biosynthesis II PWY-6872 and glutamine biosynthesis I GLNSYN-PWY (Figures 3(c)–3(e)). Panther pathway enrichment analysis results show that differential genes are mainly involved in signaling pathways (Figures 3(h) and 3(i)) such as axon guidance mediated by netrin I P00009, blood coagulation I P00011, and PI3 kinase pathway I P00048. The results of Reactome pathway enrichment analysis showed that differential genes are mainly involved in the regulation of complement cascade I R-HAS-977606, platelet activation, signaling and aggregation I R-HA S-76002, complement cascade I R-HAS-166658, and other signaling pathways (Figures 3(j) and 3(k)).

**Figure 4:**
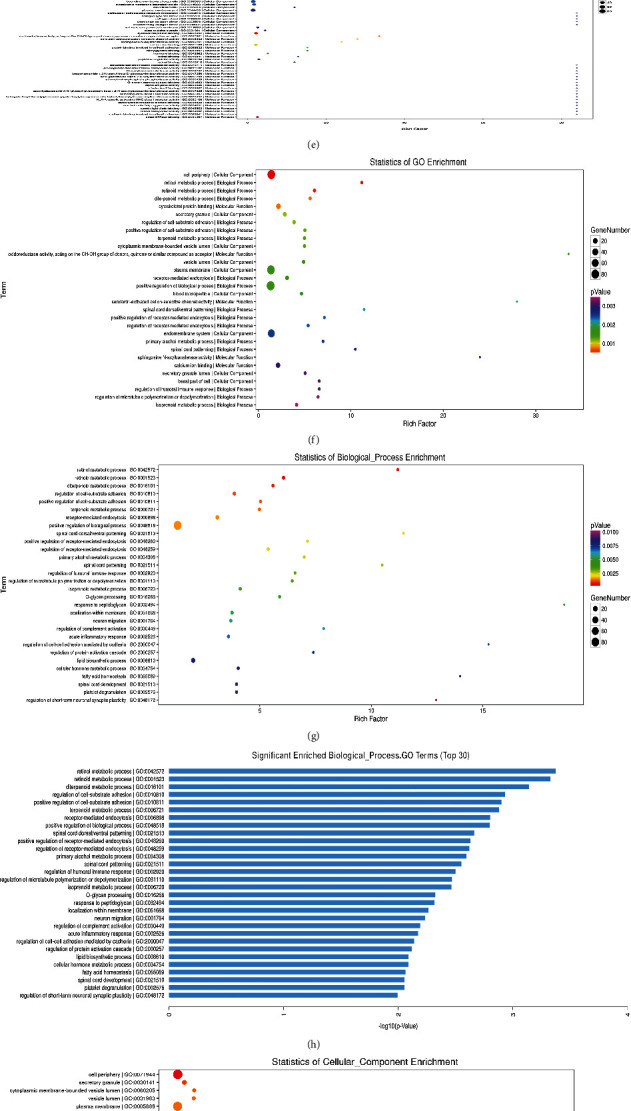
GO analysis diagram of differential genes. (a) GO standard. (b) Biological process hierarchy. (c) Cellular component hierarchy. (d) Molecular function hierarchy. (e) Significantly enriched GO terms. (f) Statistics of GO enrichment. (g) Statistics of biological enrichment. (h) Significantly enriched biological process GO terms. (i) Statistics of cellular component enrichment. (j) Significantly enriched cellular component GO terms. (k) Statistics of molecular function enrichment. (l) Significant molecular function GO terms.

*(2) GO Analysis of Differential Genes*. We mainly conduct Gene Ontology analysis on 318 differential genes from biological processes, molecular functions, and cell components. The results of biological process analysis show that the downstream differential genes regulated by TrkB are mainly involved in the regulation of retinol metabolic process, diterpenoid metabolic process, regulation of cell-substrate adhesion, and other biological processes (Figures 4(a), 4(b), 4(g), and 4(h)). The results of molecular function analysis showed that the downstream differential genes regulated by TrkB mainly affected molecular functions such as cytoskeletal protein binding, serotonin-activated cation-selective channel activity, sphingosine N-acyltransferase activity, and calcium ion binding (Figures 4(d), 4(e), 4(k), and 4(l)). Cell component analysis results show that the downstream differential genes regulated by TrkB are mainly enriched in the cell periphery, secretory granule, cytoplasmic membrane-bounded vesicle lumen, vesicle lumen, plasma membrane, blood microparticle, and other molecular components (Figures 4(c), 4(e), 4(f), 4(i), and 4(j)).

**Figure 5:**
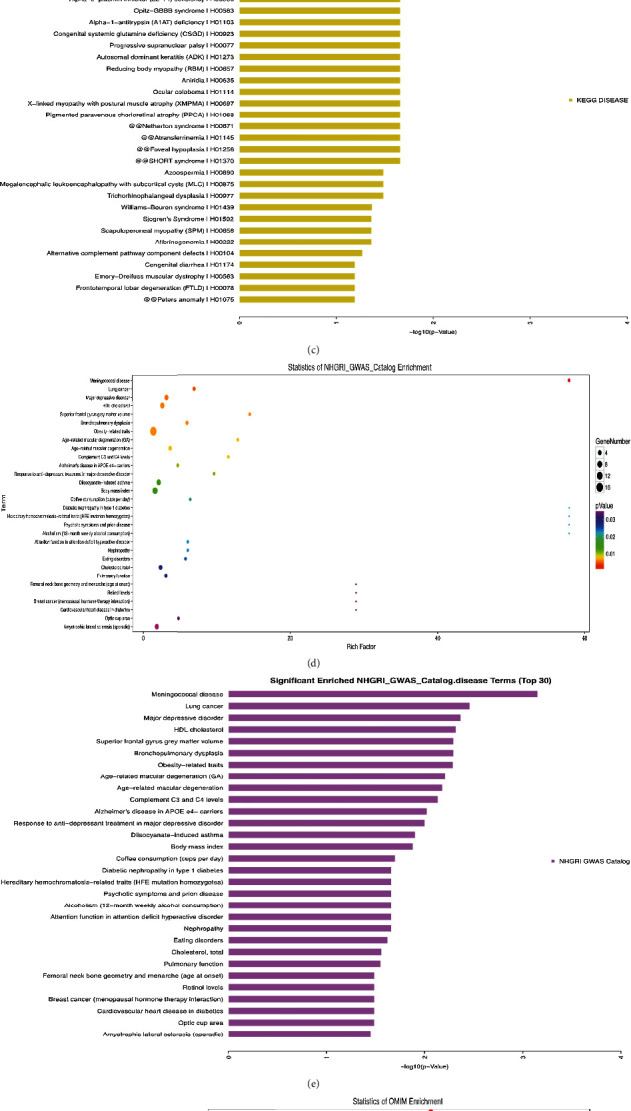
Disease enrichment analysis diagram of differential genes. (a) Statistics of disease enrichment. (b) Statistics of KEGG disease enrichment. (c) Significantly enrichment KEGG disease terms. (d) Statistics of NHGRI GWAS Catalog enrichment. (e) Significantly enriched NHGRI GWAS Catalog terms. (f) Statistics of OMIM enrichment. (g) Significantly enriched OIMI disease terms.

*(3) Disease Enrichment Analysis of Differential Genes*. After epigenetic modification of TrkB, we screened out 318 differential genes from 6 groups and performed disease enrichment analysis on these differential genes through 6 databases (OMIM, KEGG Disease, Fun DO, GAD, NHGRI, and Disease). A significantly enriched enrichment analysis chart (Figure 5) was obtained. The KEGG disease enrichment analysis results showed that the downstream differential genes regulated by TrkB are mainly involved in atypical hemolytic uremic syndrome, hematologic disease, amyotrophic lateral sclerosis, Opitz-GBBB syndrome, and other diseases (Figures 5(a)–5(c)). The results of NHGRI disease enrichment analysis showed that the downstream differential genes regulated by TrkB are mainly involved in meningococcal disease, lung cancer, major depressive disorder, HDL cholesterol, obesity-related traits, and other diseases (Figures 5(d) and 5(e)). OMIM disease enrichment analysis results show that the downstream differential genes regulated by TrkB are mainly involved in hemolytic uremic syndrome, atypical, susceptibility, asthma, inflammatory bowel disease14, emphysema due to AAT deficiency, and other diseases (Figures 5(f) and 5(g)).

**Figure 6:**
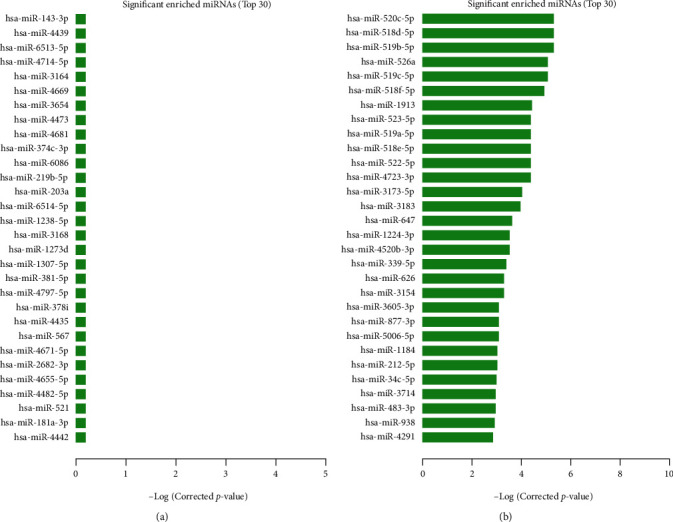
miRNA enrichment analysis diagram. (a) Significantly enriched up miRNAs (top 30). (b) Significantly enriched down miRNAs (top 30).

**Figure 7:**
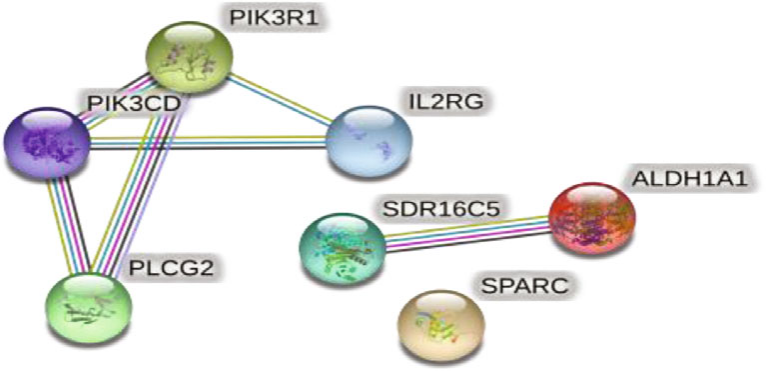
PPI network diagram of differential genes after silencing the TrkB gene in laryngeal cancer cells.

*(4) miRNA Enrichment Analysis and PPI Network*. The results of miRNA enrichment analysis showed the top 30 upregulated and downregulated miRNAs (Figures 6(a) and 6(b)). Based on the interaction data between genes in the KEGG pathway, the gene-gene interaction data within the differential gene set was screened out, and a molecular network was constructed. In Figure 7, the results show that ALDH1A1, SDR16C5, PIK3R1, PLCG2, IL2RG, PIK3CD, and SPARC are the key genes.

**Figure 8:**
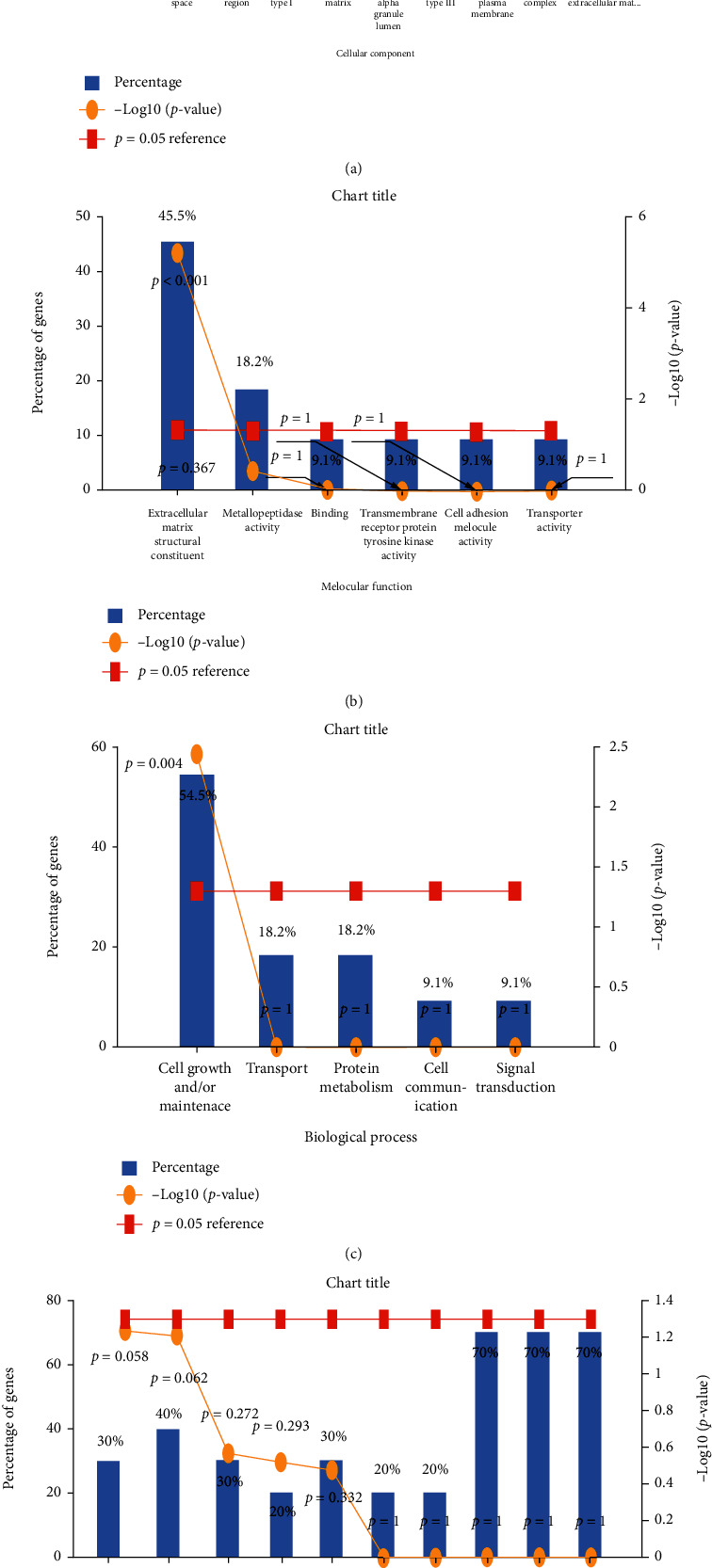
GO analysis and KEGG pathways of hub genes. (a) Biological process. (b) Cellular component. (c) Molecular function. (d) Biological pathway.

*(5) GO Analysis and KEGG Pathway of Hub Genes*. We mainly conduct Gene Ontology analysis on 7 differential genes from biological processes, molecular functions, cell components, and biological pathways. The results of biological process analysis showed that the 7 hub genes were mainly involved in regulation: signal transduction, metabolism, and energy pathways. The results of cell component analysis showed that the 7 hub genes were mainly enriched in the cytoplasm and extracellular. The results of molecular function analysis showed that the 7 hub genes mainly affected molecular functions such as lipid kinase activity, phospholipase activity, and transmembrane receptor activity. The KEGG disease enrichment analysis results showed that the 7 hub genes regulated by TrkB are mainly involved in GPV1-meditated activation cascade, EPO signaling pathway, and IL-4-mediated signaling events (Figures 8(a)–8(d)).

#### 3.2.4. RT-PCR Verification Results

**Figure 9:**
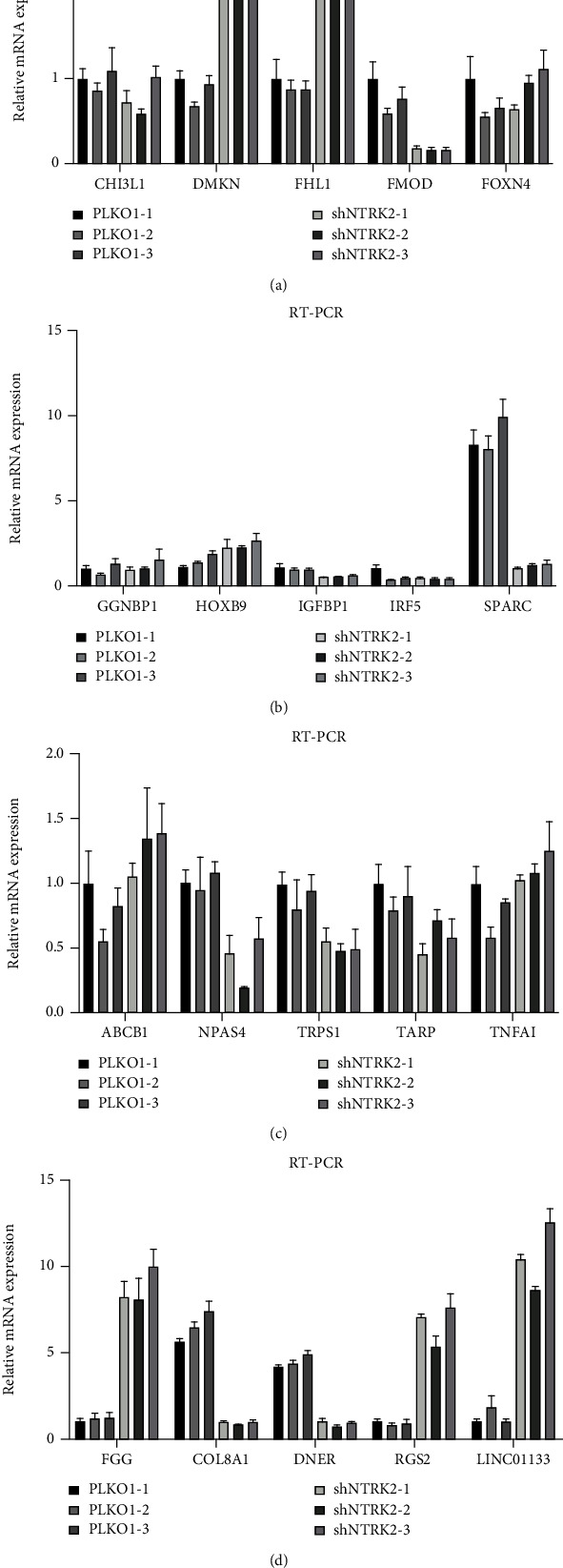
mRNA expression of key genes in experimental group and normal control group.

We extracted 10 upregulated and downregulated genes from the downstream genes regulated by TrkB and used RT-PCR to detect mRNA expression levels of differential genes. The results showed that compared with the control group, the mRNA expression levels of DMKN, FHL1, FOXN4, GGNBP1, HOXB9, ABCB1, TNFAI, RGS2, LINC01133, and FGG genes in the experimental group were significantly increased (P & LT; 0.05). Compared with the normal control group, mRNA expression levels of CHI3L1, FMOD, IGFBP1, IRF5, SPARC, NPAS4, TRPS1, TRAP, COL8A1, and DNER genes in the experimental group were significantly decreased (*P* < 0.05). All of the above confirm that the gene chip results are reliable (Figure 9).

## 4. Discussion

Laryngeal cancer is a serious threat to human health. Its early symptoms are not obvious, and it is easy to be ignored by patients. So when diagnosed, it is already in the middle or late stages. Although the current comprehensive treatment of various methods has improved the survival of patients, its clinical efficacy is not very satisfactory [19–22]; it is necessary to find new diagnoses and treatment methods to improve the prognosis and quality of life of patients. At present, the pathogenesis of laryngeal cancer has not been fully elucidated. Therefore, searching for genes that are closely related to the occurrence and development of laryngeal cancer and targeted therapy for key genes has become a popular research direction.

TrkB belongs to the family of neurotrophic factors and plays an important role in the occurrence and development of the nervous system and malignant tumors [20, 21]. The TrkB/BDNF signaling pathway is also involved in a variety of physiological functions such as tumor angiogenesis, proliferation, metastasis, and invasion and is closely related to the invasion and metastasis of a variety of malignant tumors [23]. Immunohistochemical results showed that the expression of TrkB and its ligand brain-derived neurotrophic factor (BDNF) in endometrial cancer was significantly increased (*P* < 0.05), and its high level of TrkB was associated with lymph node metastasis and lymphatic vessels Interstitial involvement is closely related (*P* < 0.05). Knockdown mediated by stable shRNA depletes TrkB and reduces the migration and invasion ability of cancer cell lines in vitro, leading to anoikic in suspension cells. In addition, overexpression of TrkB or BDNF stimulation leads to changes in the expression of molecular mediators of epithelial-mesenchymal transition (EMT). RNA interference- (RNAi-) mediated downstream regulator Twist depletion prevents TrkB-induced EMT-like transformation [24]. TrkB may become a potential Treatment target for endometrial cancer.

Preliminary studies of this group also showed that TrkB is abnormally expressed in patients with laryngeal cancer and is significantly related to the prognosis of patients. Both in vivo and in vitro experimental results show that the expression of TrkB mRNA in human laryngeal cancer Hep2 cells is significantly increased, so in this study, Hep2 cells were used for subsequent experiments. Studies have shown that downregulating TrkB can inhibit tumor growth and promote tumor cell apoptosis. TrkB can also mediate the PI3K/AKT signaling pathway to activate EMT by upregulating the expression of Twist-1 and Twist-2, thereby affecting the metastasis of laryngeal cancer [25, 26]. However, the mechanism of TrkB in laryngeal cancer has not yet been fully elucidated, and further research is needed.

In this study, human gene chip expression profiling technology was used to screen the Hep2 TrkB shRNA cell line for differential genes. A total of 318 differential genes were obtained, of which 78 were upregulated and 231 were downregulated. To verify the gene chip results, we selected 10 upregulated genes and 10 downregulated genes for RT-PCR verification. The results showed that all gene expression trends were consistent with the gene chip results, indicating that TrkB can regulate the expression of downstream genes in laryngeal cancer. The gene chip technology is accurate and can be used to study the physiological mechanism of tumors. We also used bioinformatics technology to perform GO, KEGG, and PPI enrichment analyses on the differential genes that were screened out. The signal pathway enrichment analysis showed that differential genes are mainly involved in signal transduction, global and overview maps, retinoate biosynthesis II PWY-6872, axon guidance mediated by netrin I P00009, regulation of complement cascade I R-HAS-977606, and other signal pathways. GO analysis results show that the downstream differential genes regulated by TrkB are mainly involved in the regulation of retinol metabolic process, diterpenoid metabolic process, regulation of cell-substrate adhesion, and other biological processes; the downstream differential genes regulated by TrkB mainly affect cytoskeletal protein binding, serotonin-activated cation-selective channel activity, sphingosine N-acyltransferase activity, calcium ion binding, and other molecular functions; downstream differential genes regulated by TrkB are mainly enriched in the cell periphery, secretory granule, cytoplasmic membrane-bounded vesicle lumen, vesicle lumen, plasma membrane, blood microparticle, and other molecular components. The results of disease enrichment analysis showed that the downstream differential genes regulated by TrkB are mainly involved in atypical hemolytic uremic syndrome, hematologic disease, meningococcal disease, lung cancer, major depressive disorder, hemolytic uremic syndrome, atypical, susceptibility, asthma, and other diseases. The results of miRNA enrichment analysis showed the top 30 upregulated and downregulated miRNAs. The PPI network diagram results showed that ALDH1A1, SDR16C5, PIK3R1, PLCG2, IL2RG, PIK3CD, and SPARC are the key genes. Then, we used GO analysis and KEGG enrichment analysis to see the biological process, cell component, molecular functions, and biological pathways.

The GO analysis of biological process analysis results showed that the 7 hub genes were mainly involved in regulation: signal transduction, metabolism, and energy pathway. Cell component analysis showed that the 7 hub genes were mainly enriched in cytoplasm and extracellular. The results of molecular function analysis showed that the 7 hub genes mainly affected molecular functions such as lipid kinase activity, phospholipase activity, and transmembrane receptor activity. The KEGG disease enrichment analysis results showed that the 7 hub genes regulated by TrkB are mainly involved in the GPV1-meditated activation cascade, EPO signaling pathway, and IL-4-mediated signaling events.

In this study, we used gene chip technology to screen out the downstream differential genes regulated by TrkB, explored the pathways and diseases of differential gene enrichment through bioinformatics technology, and screened out key genes through the construction of a PPI network, providing a potential therapeutic target for laryngeal cancer.

## Data Availability

The data used to support the findings of this study are included within the article.

## Abbreviations

TrkB: Tyrosine kinase receptor B
FBS: Fetal bovine serum
RT-PCR: Reverse transcription-polymerase chain reaction
BDNF: Brain-derived neurotrophic factor
EMT: Epithelial-mesenchymal transition
RNAi: RNA interference.
